# Parameters predicting [^18^F]PSMA-1007 scan positivity and type and number of detected lesions in patients with biochemical recurrence of prostate cancer

**DOI:** 10.1186/s13550-021-00783-w

**Published:** 2021-04-30

**Authors:** Niloefar Ahmadi Bidakhvidi, Annouschka Laenen, Sander Jentjens, Christophe M. Deroose, Koen Van Laere, Liesbeth De Wever, Cindy Mai, Charlien Berghen, Gert De Meerleer, Karin Haustermans, Steven Joniau, Wouter Everaerts, Karolien Goffin

**Affiliations:** 1 Department of Nuclear Medicine, University Hospitals Leuven, Herestraat 49, 3000 Leuven, Belgium; 2 Interuniversity Institute for Biostatistics and Statistical Bioinformatics, 3000 Leuven, Belgium; 3 Nuclear Medicine and Molecular Imaging, Department of Imaging and Pathology, KU Leuven, 3000 Leuven, Belgium; 4 Department of Radiology, University Hospitals Leuven, 3000 Leuven, Belgium; 5 Department of Radiation Oncology, University Hospitals Leuven, 3000 Leuven, Belgium; 6 Laboratory of Experimental Radiotherapy, Department of Oncology, KU Leuven, 3000 Leuven, Belgium; 7 Department of Urology, University Hospitals Leuven, 3000 Leuven, Belgium; 8 Urogenital, Abdominal and Plastic Surgery, Department of Development and Regeneration, KU Leuven, 3000 Leuven, Belgium

**Keywords:** [^18^F]PSMA-1007-PET/CT, Biochemical recurrence, Lesion-type, Prostate cancer, PSA

## Abstract

**Background:**

Detection of the site of recurrence using PSMA-PET/CT is important to guide treatment in patients with biochemical recurrence of prostate cancer (PCa). The aim of this study was to evaluate the positivity rate of [^18^F]PSMA-1007-PET/CT in patients with biochemically recurrent PCa and identify parameters that predict scan positivity as well as the type and number of detected lesions. This monocentric retrospective study included 137 PCa patients with biochemical recurrence who underwent one or more [^18^F]PSMA-1007-PET/CT scans between August 2018 and June 2019. PET-positive malignant lesions were classified as local recurrence, lymph node (LN), bone or soft tissue lesions. The association between biochemical/paraclinical parameters, as PSA value, PSA doubling time, PSA velocity, Gleason score (GS) and androgen deprivation therapy (ADT), and scan positivity as well as type and number of detected lesions was evaluated using logistic regression analysis (binary outcomes) and Poisson models (count-type outcomes).

**Results:**

We included 175 [^18^F]PSMA-1007-PET/CT scans after radical prostatectomy (78%), external beam radiation therapy (8.8%), ADT (7.3%), brachytherapy (5.1%) and high intensity focused ultrasound (0.7%) as primary treatment (median PSA value 1.6 ng/ml). Positivity rate was 80%. PSA value and PSA velocity were significant predictors of scan positivity as well as of the presence of bone and soft tissue lesions and number of bone, LN and soft tissue lesions, both in uni- and/or multivariable analysis. Multivariable analysis also showed prior ADT as predictor of bone and soft tissue lesions, GS as predictor of the number of bone lesions and ongoing ADT as predictor of the number of LN lesions.

**Conclusion:**

[^18^F]PSMA-1007-PET/CT showed a high positivity rate in patients with biochemically recurrent PCa. PSA value and PSA velocity were significant predictors of scan positivity as well as of the presence and number of bone and soft tissue lesions and the number of LN lesions. Our findings can guide clinicians in optimal patient selection for [^18^F]PSMA-1007-PET/CT and support further research leading to the development of a prediction nomogram.

**Supplementary Information:**

The online version contains supplementary material available at 10.1186/s13550-021-00783-w.

## Background

Prostate cancer (PCa) is a frequently diagnosed cancer worldwide, leading to a major burden of morbidity and mortality in men [1]. Biochemical recurrence of PCa after primary treatment with curative intent occurs in 30–50% of patients [2]. If this biochemical recurrence is caused by metastatic disease, systemic therapy using androgen deprivation therapy (ADT) is still considered as a corner stone in the clinical management of these patients; however it can lead to toxicity. Recent use of hybrid imaging modalities has made it possible to differentiate between local recurrence or metastatic disease, hereby offering the possibility to determine the exact number of metastatic lesions. Indeed, treating patients presenting with low-volume PCa (oligometastatic PCa) using metastasis-directed therapy, such as surgery or stereotactic body radiotherapy, is gaining interest worldwide [3–5]. Over the last few years, [^68^Ga]Ga-PSMA-11-PET/CT has replaced choline-PET/CT imaging in patients with biochemically recurrent PCa [6] and has been included in the European Association of Urology (EAU) guidelines in this setting [7]. [^68^Ga]Ga-PSMA-11-PET/CT is known for its high overall detection efficacy of 63–95%, which increases with rising serum PSA levels [8–12]. Several studies have shown that PSA, ADT and Gleason score can predict [^68^Ga]Ga-PSMA-11-PET/CT scan positivity [10, 12–16]. More recently, fluorine-18 labelled PSMA ligands have been introduced, representing several beneficial characteristics over gallium-68: fluorine-18 has a longer half-life, can be produced in large quantities in a cyclotron and has a potentially better imaging resolution due to lower positron energy [17, 18]. High detection rates between 60 and 95% have also been described for these fluorine-18 labelled PSMA ligands, such as [^18^F]PSMA-1007 and [^18^F]DCFPyL [17, 19–24]. Contrary to [^68^Ga]Ga-PSMA-11, which is excreted via urinary system, [^18^F]PSMA-1007 has the additional advantage that it is primarily excreted via the hepatobiliary system, ultimately leading to better visualization of local recurrences of PCa in proximity to the bladder [20]. In order to help the clinician in selecting suitable candidates for [^18^F]PSMA-1007-PET/CT imaging in the setting of biochemical recurrence and avoid negative and therefore unhelpful scans, it is important to identify predictors of a positive [^18^F]PSMA-1007-PET/CT scan [14]. To our knowledge, these predictors have not been previously identified in a large group of patients with biochemically recurrent PCa. The aim of this study was therefore to evaluate diagnostic performance of [^18^F]PSMA-1007-PET/CT in patients with biochemically recurrent PCa and identify parameters that predict [^18^F]PSMA-1007-PET/CT scan positivity as well as the type and number of detected lesions.

## Methods

### Patients

All patients who underwent an [^18^F]PSMA-1007-PET/CT scan in routine clinical practice for biochemical recurrence after primary definitive therapy of PCa between August 2018 and June 2019 at our institution, were included in this monocentric retrospective study. Biochemical recurrence was defined as (1) two consecutive rising serum PSA values > 0.2 ng/ml in patients who had radical prostatectomy (RP), or (2) an increase of 2 ng/ml higher than the PSA nadir value after primary radiation therapy [25]. In addition, we included 7 patients with a PSA value of less than 0.2 ng/ml, the lowest PSA value included being 0.07 ng/ml. PSA doubling time and PSA velocity were calculated using the tool available on the Memorial Sloan Kettering Cancer Center website [26]. All clinical, biochemical, histological, imaging and follow-up data were collected from the electronic patient records.

### [^18^F]PSMA-1007-PET/CT imaging

Whole-body [^18^F]PSMA-1007-PET/CT was performed from the vertex to the upper thigh on a GE Discovery MI-4 PET/CT camera (GE Healthcare, Chicago, United States of America) with time-of-flight technology or a Siemens Biograph TruePoint PET/CT camera (Siemens, Erlangen, Germany). Imaging was started 81 ± 16 min (mean ± SD) after intravenous injection of 3 MBq/kg body weight [^18^F]PSMA-1007. Briefly, [^18^F]PSMA-1007 was produced on-site using a Trasis All-in-One synthesis module, reagent kit and precursor for PSMA-1007 from ABX and fluorine-18 from an in-house cyclotron [27]. PET scan was performed for 100 s per bed position on the MI-4 PET/CT camera and 180 s per bed position on the TruePoint PET/CT camera. On the MI-4 PET/CT and TruePoint PET/CT camera [^18^F]PSMA-1007 images were reconstructed using vendor-specific software with OSEM (ordered-subset expected maximization with 3 iterations and 21 subsets) iterative reconstruction and resolution recovery (Q.Clear, a Bayesian penalized likelihood (PL) reconstruction algorithm with a beta-factor to control smoothness (respectively 600 or 1250)). PET data were corrected for dead time, random coincidence events, scatter, decay and CT-based attenuation correction.

### Image analysis

[^18^F]PSMA-1007-PET/CT images were analysed by certified nuclear medicine physicians (PET and joint protocol) and radiologists (CT) in routine clinical practice. All readers had access to all clinical information including histology and imaging reports. Visual PET analysis was performed with dedicated viewing software (Hermes Europe GmbH, Hamburg, Germany). A lesion with a focal tracer uptake that was higher than local background and not compatible with physiological uptake or known pitfalls of PSMA PET [28, 29] was considered suspicious of malignancy. The PET-positive lesions were grouped into local recurrence and distant lesions. Distant lesions were subdivided into three categories: bone, lymph node and soft tissue lesions. Oligo-recurrence was defined as three or less suspicious lesions (including local recurrence). Any suspicious lesion with high focal tracer uptake was included in the statistical analysis, unless the patient had more than ten lesions in one of the three categories. In the latter case a maximum of ten lesions per category was included in the statistical analysis.

### Statistical analysis

Logistic regression analysis was used to study the predictive effect of variables on binary outcomes (PET/CT positivity and detection of oligo-recurrence, bone, (pelvic and extrapelvic) lymph node and soft tissue lesions). Results are presented as odds ratios (OR) with 95% confidence intervals. Poisson models were used to study the effect of variables on count-type outcomes (number of positive bone, soft tissue and lymph node lesions). Results are presented as incidence rate ratios (IRR) with 95% confidence intervals. Generalized estimating equations (GEE) were used for parameter estimation to account for clustering due to patients occurring more than once in the data set. A stepwise forward model building procedure was applied to construct multivariable models of independent predictors for all outcomes. All tests are two-sided, a 5% significance level is assumed for all tests. No corrections for multiplicity were performed to the exploratory nature of the study. Analyses have been performed using SAS software (version 9.4 of the SAS System for Windows). Mean and standard deviation were reported for data with a normal distribution. Median and range were described for non-normal data. For continuous predictors an OR or IRR more than (or less than) 1 means an increased (or decreased) risk with increasing predictor level. For the categorical predictors we looked at the global *p* value for any effect. For the binary predictors/pairwise tests an OR or IRR more than (or less than) 1 shows a higher (or lower) risk for the first level. We included following predictor variables in the statistical analysis: injected activity, interval between injection and start of the scan, neoadjuvant treatment, primary treatment, pathological primary tumour staging, pathologic regional lymph node staging, positive surgical margin, Gleason score, number of lymph nodes removed, positive lymph nodes, PSA value after prostatectomy, adjuvant external beam radiation therapy (EBRT), ADT prior and/or ongoing, salvage therapy (surgery and/or EBRT), PSA doubling time, PSA velocity and PSA value at the moment of the scan.

## Results

### Patients

A total of 175 [^18^F]PSMA-1007-PET/CT scans of 137 patients were included in the study. Of the 137 included patients, 35 patients had a follow-up scan and 3 patients had a second follow-up scan. Patient characteristics are shown in Table 1. The median PSA value at the moment of imaging was 1.6 ng/ml (range 0.07–429 ng/ml). A further distinction between different primary treatments showed that the median PSA value at the moment of imaging was 1.3 ng/ml (range 0.07–250) for RP, 3.2 (range 0.4–358) for EBRT, 8.4 (range 0.6–429) for ADT, 2.6 (range 0.9–57.7) for brachytherapy and 5.0 (range 4.6–5.5) for high-intensity focused ultrasound (HIFU). Of the 107 patients who underwent RP as primary treatment, 41 had a positive surgical margin, 11 had doubtful margins and in 6 patients no data on the surgical margins were available. The median number of lymph nodes removed, when performing regional lymphadenectomy during primary treatment, was 17 (range 1–59). In 13 patients who had received neoadjuvant treatment, 12 received ADT and 1 received ADT combined with chemotherapy. After their first [^18^F]PSMA-1007-PET/CT scan, additional ADT and/or salvage surgery was given to 4 patients of which a subsequent PET/CT scan was also included in the study.

**Table 1:** Patient and [^18^F]PSMA-1007-PET/CT characteristics

Characteristics	Value		Total number of available observations
Age (years)			175
Mean ± SD^a^	69 ± 8.8		
Median (range)	70 (46–88)		
Neoadjuvant treatment	13	9.5%	137
Primary treatment			137
Radical prostatectomy	107	78%	
EBRT^b^	12	8.8%	
Androgen deprivation therapy	10	7.3%	
Brachytherapy	7	5.1%	
High Intensity Focused Ultrasound	1	0.7%	
Adjuvant external beam radiation therapy	30	22%	137
Prior androgen deprivation therapy	83	61%	137
Ongoing androgen deprivation therapy	42	24%	175
Salvage therapy			137
No salvage surgery or EBRT	76	55%	
Salvage surgery	13	9.5%	
EBRT	39	28%	
Salvage surgery and EBRT	9	6.6%	
Pathologic primary tumour staging			107
pT2	44	41%	
pT3	60	56%	
pT4	3	2.8%	
Pathologic regional lymph node staging			79
pN0	57	72%	
pN1	22	28%	
Positive surgical margin at radical prostatectomy	41	41%	101
Number of positive lymph nodes removed at primary treatment			78
Median (range)	0 (0–18)		
Gleason score			136
5	1	0.7%	
6	9	6.6%	
7	67	49%	
8	33	24%	
9	26	19%	
PSA value after radical prostatectomy (ng/ml)			102
Mean ± SD	0.4 ± 1.6		
Median (range)	0.04 (0–12)		
PSA value at PET scan (ng/ml)			175
Mean ± SD	11.1 ± 47.5		
Median (range)	1.6 (0.07–429)		
PSA doubling time (months)			171
Mean ± SD	9.5 ± 10.1		
Median (range)	6.7 (0.8–96.9)		
PSA velocity (ng/ml/month)			171
Mean ± SD	1.6 ± 9.8		
Median (range)	0.1 (0–114.5)		

^a^Standard deviation

^b^External beam radiation therapy

### [^18^F]PSMA-1007-PET/CT positivity

**Fig. 1:**
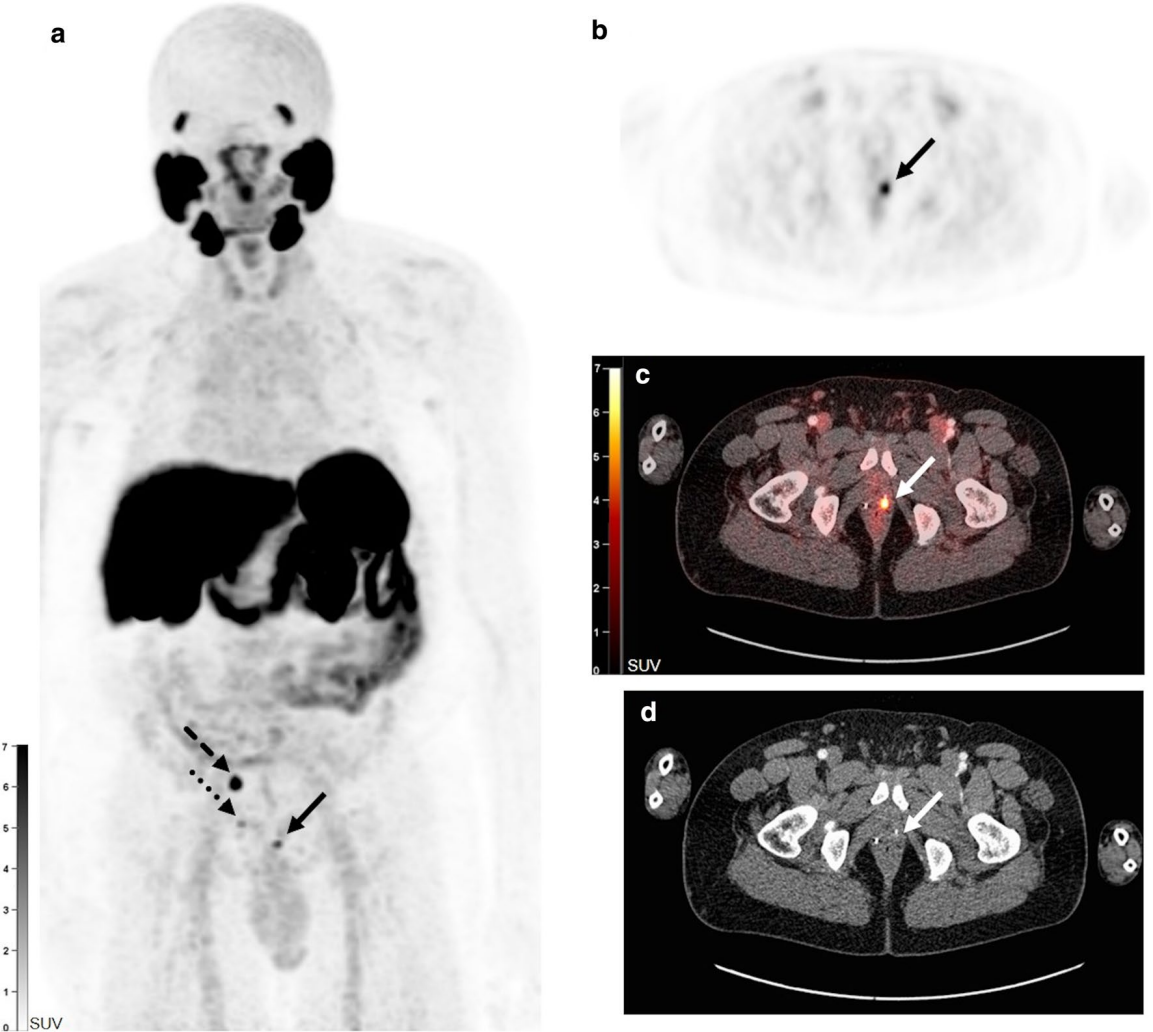
[^18^F]PSMA-1007-PET/CT scan (MIP image (**a**) and transversal PET (**b**), fusion (**c**) and CT images (**d**)) in a 64-year old patient with a medical history of radical prostatectomy with extended lymphadenectomy (Gleason score 9, pT3bN0). PSA value at the moment of [^18^F]PSMA-1007-PET/CT scan was 2.27 ng/ml. A local recurrence was detected in the prostatectomy fossa on the left side (**a**–**d**, full arrow) and in the right seminal vesicle (**a**, dotted arrow). Also, a malignant lymph node was visible at the right internal iliac artery (**a**, dashed arrow). The patient was treated with salvage external beam radiation therapy and androgen deprivation therapy with subsequent decline of PSA to 0.01 ng/ml 7 months later

One hundred and forty of 175 PET scans (80%) were positive, showing local recurrence in 40 (23%), lymph node lesions in 76 (43%), bone lesions in 58 (33%) and soft tissue lesions in 19 scans (11%) (Additional file 1: Table S1). Figure 1 shows a typical example of a patient with visualization of local recurrence. In total, 569 lesions were identified on the [^18^F]PSMA-1007-PET/CT scans, of which 203 bone lesions, 303 lymph node lesions and 63 soft tissue lesions. The maximum number of included lesions, as defined as 10 by protocol, was exceeded for bone lesions in 10 scans, for lymph node lesions in 17 scans and for soft tissue lesions in 3 scans. As mentioned, we included patients who received follow-up scans due to increasing biochemical recurrence. Two patients had an initial negative scan which turned positive in follow-up. Eighteen patients had progressive disease and six patients had stable disease on their follow-up scans. Two patients had a decrease in lesions between the initial and follow-up scan. In nine patients a persistent negative scan was seen. We did not observe a positive scan turning negative in follow-up.

### Predictors of overall [^18^F]PSMA-1007-PET/CT scan positivity and oligo-recurrence

In the univariable analysis, PSA value at the moment of the scan was a significant predictor of scan positivity (OR 1.58, *p* = 0.04) (Table 2). Also, PSA velocity was identified as a significant predictor of scan positivity (OR 67.6, *p*  = 0.03). There were no significant associations between a positive PET result and the other investigated parameters. No multivariable model could be constructed for predictors of overall scan positivity. Adjuvant EBRT was a significant predictor of oligo-recurrence and salvage therapy (including EBRT or surgery) was associated with a lower presence of oligo-recurrence (OR 0.33, *p* = 0.0009), in univariable analysis (Additional file 1: Table S2). No multivariable model could be constructed for the predictors of oligo-recurrence.

**Table 2:** Logistic regression analysis of parameters predicting overall [^18^F]PSMA-1007-PET/CT scan positivity: univariable analysis

Parameters	Test	Odds ratio (95% CI^a^); *p* value
Injected activity	+ 1 unit	1 (0.99–1.01); 0.66
Interval injection-start scan	+ 1 min	1.01 (0.99–1.04); 0.43
Neoadjuvant treatment	Yes versus no	4.39 (0.61–31.75); 0.14
Primary treatment	EBRT^b^ versus RP^c^	3.52 (0.43–29.07); 0.24
Pathological primary tumour staging	+ 1 stage	1.26 (0.53–3); 0.61
Pathological regional lymph node staging	pN1 versus pN0	4.64 (0.89–24.16); 0.07
Positive surgical margin	Global test	*p* value = 0.43
Gleason score	+ 1 level	1.2 (0.82–1.76); 0.35
Lymph nodes removed	Yes versus no	0.38 (0.14–1.08); 0.07
Number lymph nodes removed	+ 1 lymph node	1.02 (0.98–1.08); 0.33
Positive lymph nodes	Yes versus no	3.96 (0.74–21.05); 0.11
PSA value RP	+ 1 unit	4.96 (0.35–69.85); 0.24
Adjuvant EBRT	Yes versus no	1.33 (0.43–4.18); 0.62
Prior ADT^d^	Yes versus no	1.6 (0.64–4.02); 0.32
Ongoing ADT	Yes versus no	2.16 (0.58–8.04); 0.25
Salvage therapy	Yes versus no	0.63 (0.25–1.57); 0.32
PSA doubling time months	+ 1 month	0.98 (0.94–1.02); 0.26
PSA velocity	+ 1 unit	67.57 (1.56–2930.5); 0.03
PSA value	+ 1 unit	1.58 (1.02–2.45); 0.04

^a^Confidence interval

^b^External beam radiation therapy

^c^Radical prostatectomy

^d^Androgen deprivation therapy

### Predictors of type of lesion

Additional file 1: S3 and S4 show the parameters predicting the type of positive lesion on [^18^F]PSMA-1007-PET/CT. We investigated the effect of different types of primary treatment on the presence of local recurrence. Univariable analysis revealed that ADT as primary treatment was associated with higher prevalence of local recurrence compared to RP (OR 14.1, *p* = 0.0004). In addition, RP was associated with a lower prevalence of local recurrence compared to EBRT and brachytherapy (OR 0.14, *p* = 0.004 and OR 0.08, *p* = 0.007, respectively). Univariable analyses also showed that Gleason score, prior and ongoing ADT, PSA velocity and PSA value at moment of the scan were significant predictors of bone lesions. Prior ADT (OR 3.41, *p* = 0.005) and PSA value at moment of the scan (OR 1.007, *p* = 0.04) were identified as significant independent predictors of bone lesions in multivariable analysis as well. Gleason score, prior ADT, PSA velocity and PSA value were significantly correlated with the presence of soft tissue lesions on PET in univariable analysis. Additionally, multivariable analysis confirmed that prior ADT (OR 5.43, *p* = 0.02) and PSA velocity (OR 1.03, *p*  = 0.02) remained significant independent predictors of the detection of soft tissue lesions. There were no significant predictors of the presence of lymph node lesions. A further distinction between pelvic and extrapelvic lymph nodes was made (Additional file 1: Table S5). A higher pathological regional lymph node stage and higher number of lymph nodes removed at primary treatment were significantly associated with the presence of extrapelvic lymph nodes, in univariable analysis. No multivariable model could be constructed for the predictors of extrapelvic lymph node lesions. There were no significant predictors of the presence of pelvic lymph node lesions.

### Predictors of number of positive lesions

PSA velocity and PSA value were significantly correlated with the number of bone, lymph node and soft tissue lesions in univariable analysis (Additional file 1: Table S6). Multivariable analysis confirmed PSA value as an independent predictor of the number of bone lesions (IRR 1.003, *p* = 0.0002). In addition, PSA velocity was an independent predictor of the number of soft tissue lesions (IRR 1.03, *p*  < 0.001). In multivariable analysis, Gleason score and PSA value after RP were also revealed as significant independent predictors of the number of bone lesions. In addition, a higher pathological primary tumour staging was significantly correlated with a lower number of bone lesions in multivariable analysis. PSA value after RP, ongoing ADT and salvage therapy were significant independent predictors of the number of lymph node lesions. For the number of soft tissue lesions, the following parameters were significant independent predictors: type of primary treatment, positive surgical margins and prior ADT. In multivariable analysis, ADT as primary treatment was associated with a lower number of soft tissue lesions compared to RP (IRR 0.05, *p*  = 0.01).

## Discussion

Promising studies have been published about the detection of lesions in biochemically recurrent PCa with [^18^F]PSMA-1007-PET/CT, showing high detection rates between 60 and 95% [20–24]. In our study, we aimed to evaluate the diagnostic performance of [^18^F]PSMA-1007-PET/CT as well as to identify parameters that independently predict [^18^F]PSMA-1007-PET/CT scan positivity as well as the type and number of detected lesions [30].

We found an overall positivity rate of 80% for [^18^F]PSMA-1007 PET/CT. This result is in line with the study of Giesel et al., in which a detection efficacy of 81.3% was found in 251 patients with biochemical recurrence of PCa after RP and comparable PSA levels (median PSA 1.2 ng/ml) [21]. Rahbar and coworkers showed a detection efficacy of 95% in 100 patients with biochemical relapse and a median PSA level of 1.34 ng/ml [23]. It must be noted that in their study, patients were scanned 120 min after tracer injection, compared to a mean interval of 81 ± 16 min (mean ± SD) in our study. It is known for [^68^Ga]Ga-PSMA-11 as well as [^18^F]PSMA-1007 that a longer uptake time is linked to an increase in tumour-to-background ratio, thereby possibly increasing detection rates [24, 31–33]. Other studies with smaller patient cohorts have reported detection rates between 60 and 75% at median PSA levels ranging from 0.6 to 1.2 ng/ml [20, 22, 24]. Furthermore, of the 137 included patients included in our study, 35 patients had a follow-up scan and 3 patients had a second follow-up scan. The largest part of these patients had progressive disease on their follow-up scan. However, in two patients we observed a decrease in disease burden between the initial and follow-up scan due to receiving radiation therapy on the bone lesions.

In our study local recurrence was seen in 23% of the scans. A systematic review and meta-analysis by von Eyben et al., reported a local recurrence of 14% on [^68^Ga]Ga-PSMA-11-PET/CT in patients with biochemical recurrence of prostate cancer after primary treatment [34]. Our higher local detection rate is possibly due to the fact that [^18^F]PSMA-1007 is primarily excreted via the hepatobiliary system, ultimately leading to better visualization of local recurrences of PCa in proximity to the bladder [20].

Our data showed that PSA velocity and PSA value at the moment of the scan are significant predictors of [^18^F]PSMA-1007-PET/CT scan positivity (OR 67.6 and 1.6, respectively). Similarly, Witkowska-Patena et al. described PSA value as a significant predictor for [^18^F]PSMA-1007-PET/CT positivity in a cohort of 40 patients who underwent radical treatment and had low rising PSA (0.008 to ≤ 2.0 ng/ml) [22]. PSA value has also been described as a significant predictor of a positive [^68^Ga]Ga-PSMA-11-PET/CT in multiple studies [10, 13–15]. Moreover, our results show that PSA value is also a significant independent predictor of the presence and the number of bone lesions (OR 1.007 and IRR 1.003, respectively) in multivariable analysis. There are only a few studies that have investigated the influence of various parameters on the detection and number of bone metastases on [^68^Ga]Ga-PSMA-11-PET/CT. The group of Heidelberg has shown that PSA value was significantly correlated with several [^68^Ga]Ga-PSMA-11-associated parameters of bone metastases, such as the degree of tracer uptake [35]. Verburg et al. performed a retrospective study on [^68^Ga]Ga-PSMA-11-PET/CT in 155 patients with recurrent disease and reported a similar detection rate of bone metastases (32%, compared to 33% in our study) [36]. They identified that the proportion of patients with bone metastases increased with increasing PSA values, which is in accordance with the finding in our study. Additionally, PSA doubling time was the only independent predictor of bone metastases in their study. Also, Ceci and coworkers found that PSA doubling time was an independent predictor of the presence of bone metastases [37]. We could not identify PSA doubling time as a predictor of bone lesions, possibly because in patients with low PSA levels, PSA doubling time offers very little advantage, because slight changes in low PSA levels can have a large influence on the PSA doubling time value [12, 38]. Our study did, however, identify PSA velocity, which is also a parameter of PSA kinetics, as a significant predictor of the detection and number of bone lesions in univariable analysis. In addition, our study did not reveal PSA doubling time as a significant predictor of scan positivity or type or number of lymph node or soft tissue lesions. The difference in significancy between PSA doubling time and PSA velocity in our study most likely lies in the distribution of both parameters. PSA velocity has more compact values with a few strong outliers compared to PSA doubling time, leading to a more skewed distribution. Therefore, we hypothesize that PSA velocity has a more linear correlation with the outcome parameters compared to PSA doubling time.

We must take into account that previous studies have proposed that [^18^F]PSMA-1007-PET/CT exhibits a higher rate of unspecific focal bone marrow uptake [39].The group of Dietlein also saw this phenomenon when performing an intra-individual comparative study of [^18^F]PSMA-1007-PET/CT against 3 renal excreted PSMA‐tracers ([^68^Ga]Ga-PSMA-11, [^18^F]DCFPyL or [^18^F]JK‐PSMA‐7) [40]. Although the readers in our study were already aware of this drawback and other potential pitfalls of bone uptake [41], we still advice to carefully assess [^18^F]PSMA-1007 uptake in bone. A systematic approach when interpreting PSMA PET studies, for example PSMA-RADS or recently published E-PSMA standardized reporting guidelines, can be helpful when reporting these unspecific findings [42, 43].

Furthermore, we did not find a significant association between prior or ongoing use of ADT and [^18^F]PSMA-1007-PET/CT scan positivity. Conversely, our study did identify prior ADT as a significant independent predictor of the presence of bone and soft tissue lesions and the number of soft tissue lesions. In addition, ongoing ADT was identified as a significant independent predictor of the number of lymph node lesions. We did not exclude patients based on first, second or higher line ADT exposure. A previous retrospective study with [^18^F]PSMA-1007 in 251 patients with biochemical recurrence has shown a higher rate of detection in patients with prior ADT exposure [21]. It must be noted however that in this study, patients who had received second-line ADT or chemotherapy were excluded. Ongoing ADT has also been described as a significant predictor of a positive [^68^Ga]Ga-PSMA-11-PET/CT scan in multiple previous studies [8, 10, 13, 15]. It has been shown preclinically and clinically that ADT can increase PSMA expression in prostate cancer cells and that it can increase the number of lesions visualized by PSMA-PET [44, 45]. But one should be cautious about how to interpret these results, as part of the effect may also be caused by the fact that patients who underwent ADT are more likely to have more advanced PCa than those who did not. Furthermore, multiple in vitro and vivo studies investigated the temporal relationship between the initiation of ADT and PSMA uptake. In our study, the initiation of ADT varied from 3 to 220 months prior to [18F]PSMA-1007 PET/CT scan, which can be considered long-term. Predominantly, it is proposed that short-term ADT use increases PSMA uptake and long-term ADT decreases it [46, 47].

Moreover, the Gleason score has been identified as a predictor of a [^68^Ga]Ga-PSMA-11 scan positivity in several studies [12, 16]. However in our study, Gleason score was only a significant independent predictor of the number of bone lesions on [^18^F]PSMA-1007-PET/CT and not of the overall scan positivity. To the best of our knowledge, this finding has not been described in other studies with [^18^F]PSMA-1007. Further studies with larger patient cohorts are needed to validate our findings.

We investigated the effect of different types of primary treatment on the presence of local recurrence on [^18^F]PSMA-1007 PET/CT. Our study showed that ADT, EBRT and brachytherapy compared to RP were significant predictors of local recurrence on [^18^F]PSMA-1007-PET/CT scan. To our knowledge, primary treatment as a significant predictor of local recurrence on [^18^F]PSMA-1007 or [^68^Ga]Ga-PSMA-11-PET/CT has not been described previously. Our results on primary treatment as predictive parameter must be interpreted cautiously, as a part of the patients who received RP as primary treatment received adjuvant and/or salvage EBRT afterwards, making adjuvant and/or salvage EBRT a potential confounder in these findings. In accordance to our results, a study with recurrent disease on [^68^Ga]Ga-PSMA-11-PET/CT did not find a significant correlation of the parameters PSA value, PSA doubling time and Gleason score to local recurrence [36].

To the best of our knowledge, this is the first study that investigated the influence of different parameters on type and number of lesions detected on [^18^F]PSMA-1007-PET/CT scan. It is useful to know whether a patient is likely to have oligo- or polymetastatic disease, because this can lead to different treatment strategies.

Our study has some limitations. Firstly, this is a retrospective, monocentric study using a patient cohort that is heterogeneous considering the primary treatment that was given to patients, in that way representing a typical patient cohort that would be evaluated during clinical routine practice. Secondly, the PSMA-expression of the primary PCa was not known. As approximately 10% of prostate cancers are PSMA negative [48], false negative scans could have confounded our sample. Thirdly, our study merely describes the presence of positive lesions, without histological validation of all lesions or clinical follow-up. In the 11 patients in whom histological validation was available, all lesions detected on [^18^F]PSMA-1007-PET/CT were proven to be recurrent PCa. The effect of [^18^F]PSMA-1007-PET/CT on patient management is very difficult to obtain given the retrospective nature of our study and the fact that potential other imaging modalities were used to define the patient management. Nevertheless, various studies have proven that [^68^Ga]Ga-PSMA-11 PET/CT can lead to a change of treatment in a large group of patients with biochemically recurring prostate cancer [49, 50]. Further prospective studies are needed to further investigate the role of [^18^F]PSMA-1007-PET/CT on patient management. Fourthly, a standardized reporting classification system for PSMA PET was not used and results of possible additional imaging ordered by clinicians after [^18^F]PSMA-1007-PET/CT in doubtful cases were not included in our analysis.

## Conclusion

[^18^F]PSMA-1007-PET/CT was positive in 80% of patients with biochemical recurrence of prostate cancer after primary treatment. PSA value and PSA velocity were significant predictors of a positive [^18^F]PSMA-1007-PET/CT scan as well as of the presence and number of bone and soft tissue lesions and of the number of lymph node lesions in uni- and/or multivariable analysis. Further prospective studies with larger patient cohorts are needed to validate our findings and eventually lead to the development of a prediction nomogram that can help in optimal patient selection for [^18^F]PSMA-1007-PET/CT.

## Supplementary Information

**Additional file 1.** Description of positive findings (localization and number) in the [^18^F]PSMA-1007-PET/CT scans. Logistic regression analysis of the parameters predicting the presence and number of soft tissue, bone and (pelvic and extrapelvic) lymph node lesions on [^18^F]PSMA-1007-PET/CT scan and the presence of oligo-recurrence and local recurrence on [^18^F]PSMA-1007-PET/CT scan.

## Data Availability

We declare the availability of data when asked.

## Abbreviations

PCa: Prostate cancer
LN: Lymph node
GS: Gleason score
ADT: Androgen deprivation therapy
RP: Radical prostatectomy
OR: Odds ratio
IRR: Incidence rate ratio
GEE: Generalized estimating equations
EBRT: External beam radiation therapy
HIFU: High-intensity focused ultrasound
