# Supplementary material for: Parameters predicting [18F]PSMA-1007 scan positivity and type and number of detected lesions in patients with biochemical recurrence of prostate cancer
Source: EJNMMI Res. 2021 Apr 30;11:41. doi: 10.1186/s13550-021-00783-w (PMC8087750; doi:10.1186/s13550-021-00783-w)
Supplement: Supplementary file 1 — Additional file 1. Description of positive findings (localization and number) in the [18F]PSMA-1007-PET/CT scans. Logistic regression analysis of the parameters predicting the presence and number of soft tissue, bone and (pelvic and extrapelvic) lymph node lesions on [18F]PSMA-1007-PET/CT scan and the presence of oligo-recurrence and local recurrence on [18F]PSMA-1007-PET/CT scan. [file 13550_2021_783_MOESM1_ESM.docx]

**Supplementary table 1. Description of positive findings (localization and number) in 175 [^18^F]PSMA-1007-PET/CT scans**

| **Regions** | **Number of positive scans** | **Mean number of lesions per positive scan (range)** |
| --- | --- | --- |
| Overall | 140 (80%) | 4.4 (1-25) |
| Local recurrence | 40 (23%) | / |
| Lymph node lesions | 76 (43%) | 2.2 (1-10) |
| Pelvic lymph node lesions | 53 (30%) |  |
| Extrapelvic lymph node lesions | 45 (26%) |  |
| Bone lesions | 58 (33%) | 1.5 (1-10) |
| Soft tissue lesions | 19 (11%) | 0.5 (1-10) |

**Supplementary table 2. Logistic regression analysis of parameters predicting the presence of oligorecurrence on [^18^F]PSMA-1007-PET/CT scan: univariable analysis**

| **Parameters** | **Test** | **Odds ratio (95% CI^^[[1]](#footnote-1)^^);**  **p value** |
| --- | --- | --- |
| Injected activity | +1 unit | 0.999 (0.995-1.00); 0.73 |
| Interval injection-start scan | +1 minute | 0.99 (0.97-1.01);  0.23 |
| Neoadjuvant treatment | yes vs no | 1.58 (0.50-5.01);  0.44 |
| Primary treatment | global test | p = 0.32 |
| Pathological primary tumour staging | +1 stage | 1.63 (0.84-3.15);  0.15 |
| Pathological regional lymph node staging | pN1 vs pN0 | 2.07 (0.79-5.43);  0.14 |
| Positive surgical margin | global test | p = 0.66 |
| Gleason score | +1 level | 0.95 (0.69-1.32);  0.76 |
| Number lymph nodes removed | +1 lymph node | 1.02 (0.98-1.06);  0.37 |
| Positive lymph nodes | yes vs no | 2.02 (0.76-5.41);  0.16 |
| PSA value RP^^[[2]](#footnote-2)^^ | +1 unit | 0.94 (0.73-1.21);  0.62 |
| Adjuvant EBRT^^[[3]](#footnote-3)^^ | yes vs no | 2.33 (1.03-5.29);  0.04 |
| Prior ADT^^[[4]](#footnote-4)^^ | yes vs no | 0.65 (0.34-1.25);  0.20 |
| Ongoing ADT | yes vs no | 0.65 (0.31-1.38);  0.26 |
| Salvage therapy | yes vs no | 0.33 (0.17-0.63);  0.0009 |
| PSA doubling time months | +1 month | 0.99 (0.97-1.02);  0.60 |
| PSA velocity | +1 unit | 0.81 (0.61-1.08);  0.16 |
| PSA value | +1 unit | 0.98 (0.95-1.01);  0.11 |

**Supplementary table 3. Logistic regression analysis of parameters predicting the presence of local recurrence and lymph node lesions on [^18^F]PSMA-1007-PET/CT scan: univariable analysis**

|  |  | LOCAL RECURRENCE | LYMPH NODE LESIONS |
| --- | --- | --- | --- |
| Parameters | **Test** | **OR^^[[5]](#footnote-5)^^ (95% CI^^[[6]](#footnote-6)^^);**  **p value** | **OR (95% CI);**  **p value** |
| Injected activity | +1 unit | 1 (0.99;1);  0.24 | 1.00 (0.996;1.01);  0.70 |
| Interval injection-start scan | +1 minute | 1.01(0.99;1.03);  0.40 | 1 (0.98;1.02);  0.85 |
| Neoadjuvant treatment | yes vs no | 1.99 (0.60;6.6);  0.26 | 0.37 (0.09;1.52);  0.17 |
| Primary treatment | global test | p value = 0.001 | p value = 0.51 |
| pT^^[[7]](#footnote-7)^^ | +1 stage | 1.62 (0.50;5.24);  0.42 | 0.95 (0.47;1.96);  0.9 |
| pN^^[[8]](#footnote-8)^^ | pN1 vs pN0 | 0.61 (0.14;2.63);  0.51 | 1.54 (0.54;4.34);  0.42 |
| Positive surgical margin | global test | p value = 0.52 | p value = 0.40 |
| Gleason score | +1 level | 0.79 (0.52;1.2);  0.26 | 0.90 (0.64;1.27);  0.55 |
| Number lymph nodes removed | +1 lymph node | 0.99 (0.94;1.04);  0.6 | 1.03 (0.99;1.08);  0.12 |
| Positive lymph nodes | yes vs no | 0.54 (0.12;2.35);  0.41 | 1.4 (0.49;4.02);  0.54 |
| PSA value after RP^^[[9]](#footnote-9)^^ | +1 unit | 0.59 (0.20;1.75);  0.35 | 1.09 (0.85;1.40);  0.49 |
| Adjuvant EBRT^^[[10]](#footnote-10)^^ | yes vs no | 0.38 (0.11;1.26);  0.11 | 1.25 (0.54;2.91);  0.60 |
| Prior ADT^^[[11]](#footnote-11)^^ | yes vs no | 1.11 (0.48;2.55);  0.80 | 0.61 (0.31;1.23);  0.17 |
| Ongoing ADT | yes vs no | 1.76 (0.72;4.28);  0.21 | 0.97 (0.43;2.21);  0.94 |
| Salvage therapy | yes vs no | 0.43 (0.17;1.08);  0.07 | 1.27 (0.63;2.52);  0.50 |
| PSA doubling time months | +1 month | 1.02 (0.98;1.05);  0.37 | 0.99 (0.96;1.03);  0.74 |
| PSA velocity | +1 unit | 0.87 (0.70;1.09);  0.22 | 1.03 (0.98;1.08);  0.26 |
| PSA value | +1 unit | 0.99 (0.99;1.00);  0.07 | 1.01 (0.995;1.02);  0.34 |

|  |  | BONE LESIONS | | SOFT TISSUE LESIONS | |
| --- | --- | --- | --- | --- | --- |
|  |  | **Univariable** | **Multivariable** | **Univariable** | **Multivariable** |
| Parameters | **Test** | **OR^^[[12]](#footnote-12)^^ (95% CI^^[[13]](#footnote-13)^^);**  **p value** | **OR (95% CI);**  **p value** | **OR (95% CI);**  **p value** | **OR (95% CI);**  **p value** |
| Injected activity | +1 unit | 1.00 (0.997-1.01);  0.36 |  | 1.00 (0.997-1.01);  0.26 |  |
| Interval injection-start scan | +1 minute | 1 (0.98-1.02);  0.87 |  | 1.01 (0.98-1.03);  0.53 |  |
| Neoadjuvant treatment | yes vs no | 2.50 (0.91-6.91);  0.08 |  | 1.11 (0.24-5.2);  0.9 |  |
| Primary treatment | global test | p value = 0.14 |  | p value = 0.55 |  |
| pT^^[[14]](#footnote-14)^^ | +1 stage | 0.69 (0.32-1.52);  0.36 |  | 1.19 (0.39-3.61);  0.76 |  |
| pN^^[[15]](#footnote-15)^^ | pN1 vs pN0 | 1.87 (0.65-5.36);  0.25 |  | 3.24 (0.56-18.71);  0.19 |  |
| Positive surgical margin | global test | p value = 0.91 |  | p value = 0.22 |  |
| Gleason score | +1 level | 1.45 (1.02-2.07);  0.04 |  | 1.84 (1.00-3.38);  0.049 |  |
| Number lymph nodes removed | +1 lymph node | 1.00 (0.96-1.04);  0.97 |  | 0.99 (0.89-1.09);  0.80 |  |
| Positive lymph nodes | yes vs no | 1.73 (0.59-5.05);  0.32 |  | 2.69 (0.46-15.56);  0.27 |  |
| PSA value after RP^^[[16]](#footnote-16)^^ | +1 unit | 1.22 (1-1.49);  0.05 |  | 1.08 (0.86;1.35);  0.53 |  |
| Adjuvant EBRT^^[[17]](#footnote-17)^^ | yes vs no | 0.76 (0.32-1.80);  0.53 |  | 1.15 (0.29-4.56);  0.84 |  |
| Prior ADT^^[[18]](#footnote-18)^^ | yes vs no | 3.64 (1.57-8.44);  0.003 | 3.41 (1.46-7.96);  0.005 | 6.4 (1.48-27.64);  0.01 | 5.43 (1.25-23.65);  0.02 |
| Ongoing ADT | yes vs no | 2.6 (1.18-5.7);  0.02 |  | 2.02 (0.63-6.43);  0.24 |  |
| Salvage therapy | yes vs no | 1.13 (0.55-2.30);  0.74 |  | 2.59 (0.84-8.01);  0.1 |  |
| PSA doubling time months | +1 month | 0.97 (0.89-1.06);  0.52 |  | 0.95 (0.88-1.03);  0.21 |  |
| PSA velocity | +1 unit | 1.08 (1.01-1.16);  0.04 |  | 6.00 (1.6-22.54);  0.01 | 1.03 (1.01-1.06);  0.02 |
| PSA value | +1 unit | 1.01 (1.00-1.02);  0.01 | 1.007 (1.00-1.014);  0.04 | 1.007 (1.00-1.013);  0.03 |  |

**Supplementary table 4. Logistic regression analysis of parameters predicting the presence of bone and soft tissue lesions on [^18^F]PSMA-1007-PET/CT scan: univariable and multivariable analysis**

**Supplementary table 5. Logistic regression analysis of parameters predicting the presence of pelvic and extrapelvic lymph node lesions on [^18^F]PSMA-1007-PET/CT scan: univariable analysis**

|  |  | PELVIC LYMPH NODES | EXTRAPELVIC LYMPH NODES |
| --- | --- | --- | --- |
| Parameters | **Test** | **OR^^[[19]](#footnote-19)^^ (95% CI^^[[20]](#footnote-20)^^);**  **p value** | **OR (95% CI);**  **p value** |
| Injected activity | +1 unit | 0.998 (0.99-1.00);  0.51 | 1.003 (1.00-1.01);  0.26 |
| Interval injection-start scan | +1 minute | 1.01 (0.99-1.03);  0.27 | 1.00 (0.98-1.02);  0.79 |
| Neoadjuvant treatment | yes vs no | 0.28 (0.06-1.34);  0.11 | 0.59 (0.11-3.06);  0.53 |
| Primary treatment | global test | p=0.18 | p=0.14 |
| pT^^[[21]](#footnote-21)^^ | +1 stage | 0.87 (0.41-1.85);  0.72 | 1.01 (0.45-2.28);  0.98 |
| pN^^[[22]](#footnote-22)^^ | pN1 vs pN0 | 0.41 (0.12-1.45);  0.17 | 3.33 (1.09-10.24);  0.04 |
| Positive surgical margin | global test | p=0.88 | p=0.66 |
| Gleason score | +1 level | 0.91 (0.62-1.35);  0.65 | 1.10 (0.74-1.63);  0.65 |
| Number lymph nodes removed | +1 lymph node | 0.99 (0.94-1.04);  0.71 | 1.05 (1.00-1.10);  0.04 |
| Positive lymph nodes | yes vs no | 0.41 (0.12-1.46);  0.17 | 2.67 (0.86-8.26);  0.09 |
| PSA value after RP^^[[23]](#footnote-23)^^ | +1 unit | 1.11 (0.88-1.41);  0.37 | 1.25 (0.91-1.72);  0.16 |
| Adjuvant EBRT^^[[24]](#footnote-24)^^ | yes vs no | 0.55 (0.22-1.41);  0.21 | 1.21 (0.46-3.23);  0.70 |
| Prior ADT^^[[25]](#footnote-25)^^ | yes vs no | 0.71 (0.34-1.46);  0.35 | 1.10 (0.47-2.55);  0.83 |
| Ongoing ADT | yes vs no | 1.21 (0.52-2.80);  0.66 | 1.65 (0.67-4.03);  0.28 |
| Salvage therapy | yes vs no | 1.32 (0.65-2.68);  0.44 | 1.50 (0.67-3.40);  0.33 |
| PSA doubling time months | +1 month | 1.00 (0.97-1.04);  0.90 | 0.97 (0.92-1.02);  0.20 |
| PSA velocity | +1 unit | 1.02 (1.00-1.04);  0.13 | 1.05 (0.96-1.15);  0.25 |
| PSA value | +1 unit | 1.00 (0.997-1.01);  0.31 | 1.01 (0.99-1.03);  0.25 |

|  |  | BONE LESIONS | | LYMPH NODE LESIONS | | SOFT TISSUE LESIONS | |
| --- | --- | --- | --- | --- | --- | --- | --- |
|  |  | **Univariable** | **Multivariable** | **Univariable** | **Multivariable** | **Univariable** | **Multivariable** |
| Parameters | **Test** | **IRR^^[[26]](#footnote-26)^^ (95% CI^^[[27]](#footnote-27)^^);**  **p value** | **IRR (95% CI);**  **p value** | **IRR (95% CI);**  **p value** | **IRR (95% CI);**  **p value** | **IRR (95% CI);**  **p value** | **IRR (95% CI);**  **p value** |
| Injected activity | +1 unit | 1.00 (0.996-1.01);  0.73 |  | 1.00 (0.997-1.01);  0.56 |  | 1 (0.99-1.01);  0.77 |  |
| Interval injection-start scan | +1 minute | 1.01 (0.99-1.03);  0.37 |  | 1.00 (0.99-1.02);  0.54 |  | 1.03 (1-1.05);  0.07 |  |
| Neoadjuvant treatment | yes vs no | 2.02 (0.71-5.74);  0.19 |  | 0.93 (0.28-3.12);  0.90 |  | 1.10 (0.21-5.8);  0.91 |  |
| Primary treatment | global test | p value = 0.18 |  | p value = 0.25 |  | p value = 0.92 | p value = 0.04 |
| pT^^[[28]](#footnote-28)^^ | +1 stage | 0.51 (0.23-1.13);  0.1 | 0.29 (0.14-0.61)  0.001 | 0.81 (0.43-1.56);  0.53 |  | 0.83 (0.16-4.27);  0.82 |  |
| pN^^[[29]](#footnote-29)^^ | pN1 vs pN0 | 1.14 (0.38-3.44);  0.82 |  | 1.1 (0.44-2.74);  0.84 |  | 0.89 (0.12-6.73);  0.91 |  |
| Positive surgical margin | global test | p value=0.55 |  | p value = 0.75 |  | p value = 0.15 | p value = 0.001 |
| Gleason score | +1 level | 1.46 (1.02-2.09);  0.04 | 1.57 (1.16-2.13);  0.003 | 0.94 (0.7-1.27);  0.69 |  | 2.28 (1.33-3.90);  0.003 |  |
| Number lymph nodes removed | +1 lymph node | 1.02 (0.98-1.07);  0.29 |  | 1.00 (0.97-1.03);  0.96 |  | 0.94 (0.79-1.12);  0.48 |  |
| Positive lymph nodes | yes vs no | 1.04 (0.33-3.25);  0.95 |  | 0.95 (0.38-2.38);  0.91 |  | 0.75 (0.1-5.60);  0.78 |  |
| PSA value after RP^^[[30]](#footnote-30)^^ | +1 unit | 1.2 (1.1-1.31);  <0.0001 | 1.28 (1.20-1.35);  <0.0001 | 1.08 (0.97-1.20);  0.19 | 1.15 (1.03-1.28);  0.01 | 1.16 (0.92-1.45);  0.21 |  |
| Adjuvant EBRT^^[[31]](#footnote-31)^^ | yes vs no | 0.5 (0.2-1.26);  0.14 |  | 0.70 (0.33-1.47);  0.35 |  | 0.57 (0.13-2.55);  0.46 |  |
| Prior ADT^^[[32]](#footnote-32)^^ | yes vs no | 2.50 (0.85-7.38);  0.1 |  | 1.45 (0.76-2.78);  0.26 |  | 20.83 (4.30-100.85);  0.0002 | 22.16 (3.70-132.69);  0.0007 |
| Ongoing ADT | yes vs no | 1.79 (0.81-3.99);  0.15 |  | 2.31 (1.24-4.31);  0.01 | 2.27 (1.22-4.21);  0.01 | 3.37 (0.74-15.22);  0.12 |  |
| Salvage therapy | yes vs no | 1.3 (0.6-2.82);  0.51 |  | 1.76 (0.96-3.23);  0.07 | 2.11 (1.10-4.03);  0.02 | 3.39 (0.85-13.51);  0.08 |  |
| PSA doubling time months | +1 month | 0.97 (0.89-1.06);  0.50 |  | 0.97 (0.93-1.02);  0.21 |  | 0.93 (0.86-1.01);  0.09 |  |
| PSA velocity | +1 unit | 1.022 (1.017-1.027);  <0.001 |  | 1.019 (1.013-1.024);  <0.001 |  | 1.024 (1.016-1.032);  <0.001 | 1.03 (1.02-1.05);  <0.001 |
| PSA value | +1 unit | 1.005 (1.004-1.007);  <0.001 | 1.003 (1.002-1.005);  0.0002 | 1.005 (1.003-1.007);  <0.001 |  | 1.006 (1.003-1.009);  0.0002 |  |

**Supplementary table 6. Logistic regression analysis of parameters predicting the number of positive lesions on [^18^F]PSMA-1007-PET/CT scan: univariable and multivariable analysis**

1. confidence interval [↑](#footnote-ref-1)
2. radical prostatectomy [↑](#footnote-ref-2)
3. external beam radiation therapy [↑](#footnote-ref-3)
4. androgen deprivation therapy [↑](#footnote-ref-4)
5. Odds ratio [↑](#footnote-ref-5)
6. confidence interval [↑](#footnote-ref-6)
7. pathological primary tumour staging [↑](#footnote-ref-7)
8. pathological regional lymph node staging [↑](#footnote-ref-8)
9. radical prostatectomy [↑](#footnote-ref-9)
10. external beam radiation therapy [↑](#footnote-ref-10)
11. androgen deprivation therapy [↑](#footnote-ref-11)
12. Odds ratio [↑](#footnote-ref-12)
13. confidence interval [↑](#footnote-ref-13)
14. pathological primary tumour staging [↑](#footnote-ref-14)
15. pathological regional lymph node staging [↑](#footnote-ref-15)
16. radical prostatectomy [↑](#footnote-ref-16)
17. external beam radiation therapy [↑](#footnote-ref-17)
18. androgen deprivation therapy [↑](#footnote-ref-18)
19. Odds ratio [↑](#footnote-ref-19)
20. confidence interval [↑](#footnote-ref-20)
21. pathological primary tumour staging [↑](#footnote-ref-21)
22. pathological regional lymph node staging [↑](#footnote-ref-22)
23. radical prostatectomy [↑](#footnote-ref-23)
24. external beam radiation therapy [↑](#footnote-ref-24)
25. androgen deprivation therapy [↑](#footnote-ref-25)
26. Incidence Rate Ratio [↑](#footnote-ref-26)
27. confidence interval [↑](#footnote-ref-27)
28. pathological primary tumour staging [↑](#footnote-ref-28)
29. pathological regional lymph node staging [↑](#footnote-ref-29)
30. radical prostatectomy [↑](#footnote-ref-30)
31. external beam radiation therapy [↑](#footnote-ref-31)
32. androgen deprivation therapy [↑](#footnote-ref-32)
